# Comparison of Methods for Bulk Automated Simulation of Glycosidic Bond Conformations

**DOI:** 10.3390/ijms21207626

**Published:** 2020-10-15

**Authors:** Victor Stroylov, Maria Panova, Philip Toukach

**Affiliations:** 1 N.D. Zelinsky Institute of Organic Chemistry, Russian Academy of Sciences, 119991 Moscow, Russia; victor.stroylov@gmail.com (V.S.); mariya_13-09@mail.ru (M.P.); 2 Chemical Faculty, National Research University Higher School of Economics (HSE), 20 Myasnitskaya Street, 101000 Moscow, Russia

**Keywords:** carbohydrate, disaccharide, glycosidic bond conformation, molecular dynamics, NOE simulation, database filling, nuclear Overhauser effect, force field

## Abstract

Six empirical force fields were tested for applicability to calculations for automated carbohydrate database filling. They were probed on eleven disaccharide molecules containing representative structural features from widespread classes of carbohydrates. The accuracy of each method was queried by predictions of nuclear Overhauser effects (NOEs) from conformational ensembles obtained from 50 to 100 ns molecular dynamics (MD) trajectories and their comparison to the published experimental data. Using various ranking schemes, it was concluded that explicit solvent MM3 MD yielded non-inferior NOE accuracy with newer GLYCAM-06, and ultimately PBE0-D3/def2-TZVP (Triple-Zeta Valence Polarized) Density Functional Theory (DFT) simulations. For seven of eleven molecules, at least one empirical force field with explicit solvent outperformed DFT in NOE prediction. The aggregate of characteristics (accuracy, speed, and compatibility) made MM3 dynamics with explicit solvent at 300 K the most favorable method for bulk generation of disaccharide conformation maps for massive database filling.

## 1. Introduction

The role of polysaccharides in biological phenomena, such as cell–cell interaction and inflammatory processes and immunity, can be hardly overestimated [1,2]. Oligo- and polysaccharide conformations are crucial for understanding of their biological functions. The conformation data are utilized in the studies of glycan interaction with proteins and other biomolecules, and in calculation of the macroscopic molecular parameters, such as NMR observables. There are no direct physical methods to probe conformation, while in silico determination of saccharide conformation, in general, is still an unsolved task. Despite there being plenty of molecular dynamics (MD) simulation methods, [3] the software tools in which these methods are implemented for glycans require users to be specialists in the field, and are difficult to learn for non-informaticians [4,5]. Moreover, many existing molecular mechanic force fields dedicated to carbohydrates are parameterized for a limited set of monosaccharide building blocks not covering the natural diversity of glycans, especially beyond mammalian class [6]. Our challenge is to implement the accurate saccharide conformation prediction (1) easily available to all glycochemists and glycobiologists and (2) not limited to specific structural features such as particular monomeric components of a molecule. As one of demands, there is a need for a reliable, fast, and easily available method for saccharide conformation simulation. Easy accessibility can be achieved by an automated procedure for conformation simulation in the framework of an existing glycoinformatics project. We plan usage of the Carbohydrate Structure Database (CSDB, http://csdb.glycoscience.ru) [7] as a host project and a source of data on molecular geometry.

In the majority of natural glycans, at least of those composed mainly of pyranoses, saccharide conformational behavior implies relatively stable monosaccharides occupying a few (often, one) predominant conformations and flexible *inter-*residue bridges [8]. Disaccharides are the simplest carbohydrate molecules presenting all the types of rotational degrees of freedom characteristic for oligo- and polysaccharides. This observation allows one to split a glycan molecule into pairs and triples of monosaccharides (for linear and branched nodes, respectively) and subsequently combine small fragment conformations into conformations of a bigger glycan. Such resulting conformations can be further optimized, used as initial geometries in calculations at higher levels of theory, or used as rough approximations representing a conformational ensemble of a saccharide. Moreover, disaccharides were proven to be adequate benchmark systems for simulation methodology validation in the context of subsequent usage as models for larger molecules [9].

Distribution of geometrical parameters of constituent fragments, such as sustainable monosaccharide conformations and favorable glycosidic and exocyclic linkage configurations, can be pre-calculated and stored in a database, thus increasing simulation performance for any given glycan. While the majority of natural monosaccharide residues adopt a few ring conformations, e.g., ^4^C_1_ for most D-hexapyranoses, determination of a glycosidic bond conformation is more complicated, since its populated torsion angles tend to have different values depending on a particular pair of linked monosaccharides.

In this study, we aimed at finding out a best method for quick, reliable, and maximally automated prediction of the glycosidic bond conformation in disaccharides. The criteria for the comparison are the simulation accuracy and speed, compatibility with abundant structural features, and easiness of implementation in bulk computer simulations. They follow from the planned usage of a chosen method, which is filling a sub-database of disaccharide conformation maps at CSDB platform. In the future, this database will store data on several thousands of disaccharides most abundant in natural glycopolymer and glycoconjugate sequences, as ranked by the statistical analysis of glycomes [10]. The accuracy of the methods we tested was ranked by predictions of nuclear Overhauser effects (NOEs) from each conformational ensemble and their comparison to the experimental data on a sampling of disaccharides, for which steady state NOE data were published. We also included quantum mechanical Density Functional Theory (DFT) calculations as a reference method.

## 2. Results

The reliability of the predicted conformational behavior of disaccharides can be probed by comparison of properties predicted theoretically and measured experimentally. In solution, the method of choice to study the 3D structure of saccharides is NMR. Through coupling constants, it allows one to evaluate the magnitude of the torsion angles and nuclear Overhauser effects (NOEs) and estimate distances between protons located in close proximity. For saccharides built of relatively rigid building blocks linked by flexible bonds, the key parameters are those measured through the glycosidic bonds. All these NMR parameters represent average values, as all of the rapidly interconverting conformations are accessible to the molecule on the NMR timescale due to rotational freedom of glycosidic bond torsions. This means that a straightforward interpretation of experimental data leads to the elucidation of a “virtual conformation” with only a limited physical meaning. It was reported that NOEs predicted from an average conformation showed poorer accuracy than NOEs averaged from a sampling of individual NOEs in each conformational minimum [11].

We compared a number of force fields most demanded in carbohydrate molecular simulation [12] by prediction of glycosidic bond conformation in disaccharides and validation of predictions against experimental NOEs. Four all-chemical (MM3-2000 [13], MMFF94 [14], OPLS-aa [15], and Amber ff14SB [16,17,18]) and two dedicated (GLYCAM-06 [6] and CHARMM [19,20] with C36-carb parameter set) force fields were used. We used the Tinker software package, as it was freely available, convenient for learning, and had easy features for batch programming [21]. Moreover, Conformational Analysis Tools (CAT) software exists [22] for automatic analysis of Tinker output, including generation of conformation maps from MD trajectories. It was shown that Tinker MM3 can be used to estimate carbohydrate energies and geometries accurately, and to pursue studies on the potential energy surfaces of di- and trisaccharides [23]. Amber [24] and Gromacs [25] were chosen as a well-known and popular software packages providing calculations in Amber ff14SB and carbohydrate-dedicated force fields. In cases requiring de novo molecule parameterization or manual topology manipulation, we used Gromacs software [26], as it employed convenient human-readable topology format, and produced results merely distinguishable from other simulation packages, such as Amber (see Figure 4). It should be noted that charged disaccharides pose a significant parameterization challenge in both protein and carbohydrate force fields. Even dedicated carbohydrate fields such as GLYCAM lack ready-to-use parameters for certain residues (e.g., Rhap4N; see molecules **7**, **8**, **9**, and **11**), and require topology manipulation to accomplish simulation for all molecules under study (see Table 1).

In further discussion, dihedral angles that form glycosidic bonds were defined as follows: φ = H1–C1–O–Cx (C1–C2–O–C4′ for α-Kdo-(2-4)- α-Kdo-O-Allyl), ψ = C1–O–Cx–Hx(*pro-S*) (C1–O–C2′–C1′ for α-D-Glc*p*-(1-2)-β-D-Fru*f*), and ω = H(x–1)–C(x–1)–Cx-Hx(*pro-S*). C1/C2 stands for the anomeric center in the glycosidic bond donor in aldose/ketose, respectively, and Cx stands for the substituted position in the glycosidic bond acceptor. If there are more than one Hx protons, *pro-S* one is used. If a donor and acceptor cannot be distinguished (e.g., in sucrose), the residue located at the left side in CSDB Linear encoding [27] was considered as a donor. The used hydrogen-based definition for Ramachandran plotting was pointed out as optimal for graphic representation torsional maps [28].

In order to compare force fields, eleven disaccharides were chosen as models (see details in Methods). We compared glycosidic bond torsion population from MD trajectories obtained with implicit solvation model default for each force field, and with explicit water. Conformation map minima derived from DFT calculations were used as an example of achievable accuracy. We chose NOE comparison for the experimental validation since this NMR observable depends on the *inter-*glycosidic proton distances over the whole ensemble of conformations, accounting for all torsions involved in energy distribution. Besides force field, accuracies of predicted NOE values critically depend on the quality of the configurational space sampling affected mainly by the simulation temperature and duration (number of steps). However, increasing simulation duration is often impractical due to associated increase in computational cost, especially in bulk high-throughput simulations, which are the intended applications of the method under choice. Temperature increase can lead to deterioration of conformational ensemble, since NOE relates to the conformational ensemble at the experimental temperature. We have studied the effects of both parameters (simulation duration and temperature) on the accuracy of predicted NOEs.

If a limited number of conformational states exist in an MD trajectory, NOE can be calculated as a trajectory average of individual NOEs depending on the inverse sixth power of inter-proton distance [29]. However, such trajectory averaging requires that distance variations between protons are uncorrelated, which, in turn, requires that transitions between conformational states occur much slower than molecular fluctuations. Newer approaches that calculate NOEs from trajectory-derived correlation functions exist [30]; however, they require lengthening MD trajectories even further and were not considered in this study due to inapplicability for bulk calculations.

**Figure 1:**
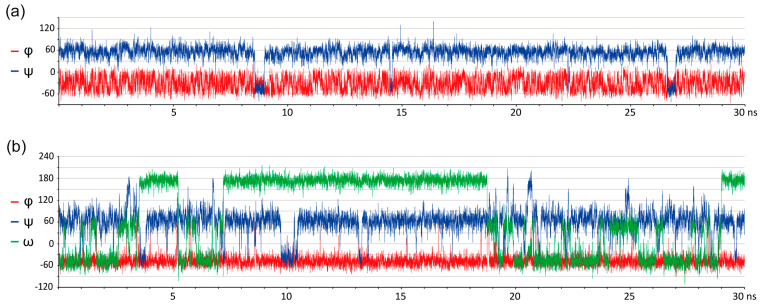
Transitions of a glycosidic bridge conformation in sucrose **1** (**a**) and 1-6-dimannose **4** (**b**) in molecular dynamics in GLYCAM force field with explicit water at 300 K. First 30 ns of 100 ns trajectory part is depicted for clarity

To begin, we took 100 ns trajectory length [31] and compared performance of each force field at 300 K, as it was the average temperature of NOE measurement, and at 1000 K, as a deliberately high temperature ensuring the appearance of all transitions and enhancing sampling statistics. To substantiate that this trajectory length is enough to model all transitions in the conformational space of glycosidic torsions, we probed dynamics of a molecule with two torsions (α-D-Glc*p*-(1-2)-β-D-Fru*f*, 1) and of one with three torsions (α-D-Man*p*-(1-6)-β-D-Man*p*-OMe, **4**). As a typical example (Figure 1a), sucrose (**1**) in GLYCAM showed time-stable φ dihedral in explicit-water dynamics, while ψ dihedral tended to occupy a local minimum for ~0.7 ns every ~15 ns. Water model did not affect location of minima but changed their relative population (**1**: ψ, GB: 56.5 ± 16.5 (88%), −47.9 ± 12.0 (11%), 168 ± 9.2 (1%); ψ, Transferable Intermolecular Potential 3 Point (TIP3P): 56.4 ± 13.5 (97.3%), −47.4 ± 12.6 (2.7%)). Implicit water dynamics showed more often transitions and shorter lifetime of conformations, as well as an additional minor minimum. This observation is in agreement with a hypothesis that disaccharide conformations are stabilized by hydrogen bonds with solvent. In a glycosidic linkage with three rotational degrees of freedom, ψ transitions were more frequent, and ω torsion (*pro-S* H6-C6-C5-H5) occupied one or more of the three stable conformations (*gg*, *gt*, and *tg*) (Figure 1b), as often happens in 1-6-linked disaccharides [32,33]. From the considerations mentioned above, we conclude that 50 ns trajectory length is acceptable for further simulations. Identification of plausible conformations fits the purpose of this study, even without achieving a truly converged MD trajectory, which can require a microsecond or millisecond timescale [34].

**Figure 2:**
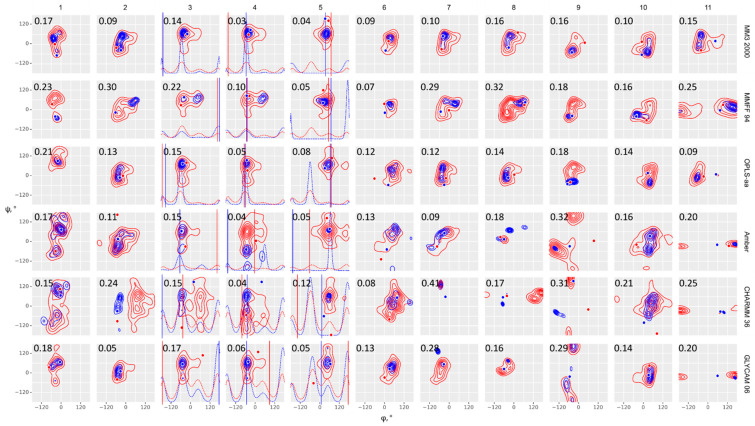
Glycosidic bond conformation plots modeled at 300 K (blue) and 1000 K (red) with implicit solvent models. Columns are eleven molecules under study; rows are force fields, as indicated on the right. Dihedrals are defined as φ = H1–C1–O–Cx (C1–C2–O–C4′ for α-Kdo-(2-4)-α-Kdo-OAllyl) and ψ = C1–O–Cx–Hx (C1–O–C2′–C1′ for α-D-Glc*p*-(1-2)-β-D-Fru*f*), ω = Cx–Cx–C(x+1)-H(*pro-S*). Blue (300 K) and red (1000 K) dots (φ, ψ) and lines (ω) stand for starting geometries after minimization (MOL files are available in Supplementary materials). The density plots were obtained from full molecular dynamics (MD) trajectories. Contour levels denote equal difference in density of sampling. Dotted lines in molecules 3, 4, and 5 denote ω angle distribution. Root mean square deviation (RMSD) between experimental and calculated NOEs averaged on all interacting protons is provided in the upper left corner of each plot.

**Figure 3:**
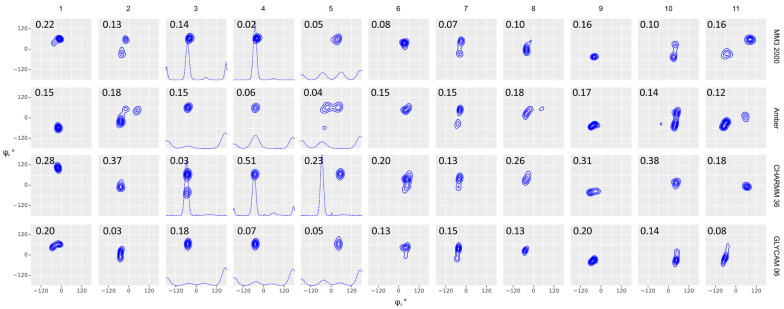
Glycosidic bond conformation plots modeled at 300 K with explicit solvent models. Columns are eleven molecules under study; rows are force fields. Dihedrals are defined as φ = H1–C1–O–Cx (C1–C2–O–C4′ for α-Kdo-(2-4)-α-Kdo link) and ψ = C1–O–Cx–Hx (C1–O–C2′–C1′ for α-D-Glcp-(1-2)-β-D-Fruf), ω = Cx–Cx–C(x+1)-H(*pro-S*). Black points (φ, ψ) and lines (ω) stand for starting geometries after minimization (MOL files are available in Supplementary materials). The density plots were obtained from full MD trajectories. Contour levels denote equal difference in density of sampling. Dotted lines in molecules 3, 4 and 5 denote ω angle distribution. RMSD between experimental and calculated NOEs averaged on all interacting protons is provided in the upper left corner of each plot.

**Figure 4:**
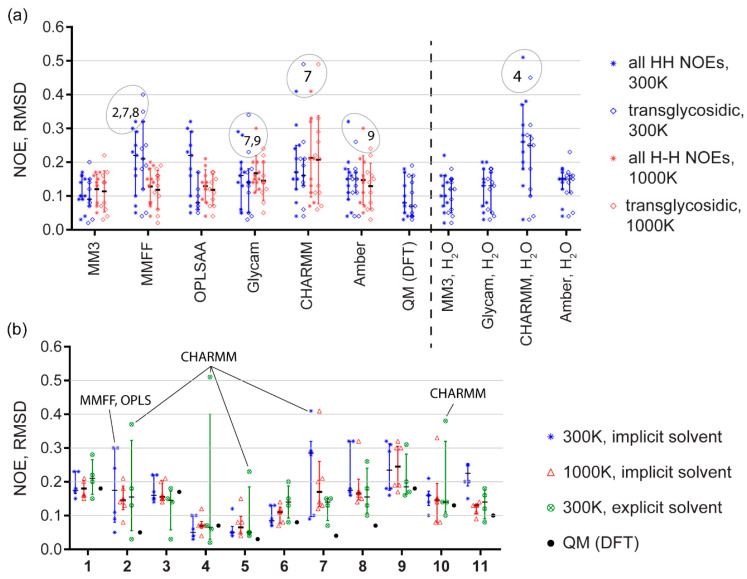
(**a**) Comparison of force fields on a sampling of eleven molecules (means; interquartile ranges). Data for 300 K (blue) and 1000 K (red) molecular dynamics are shown. MD with implicit solvation model is to the left of a dashed line, MD with explicit solvent is on the right. In each column, data for all NOEs (left bar, stars) and for transglycosidic NOEs only (right bar, diamonds) are given separately. Outliers are encircled and supplied with molecule numbers. (**b**) Comparison of model structures in regard to NOE predictive power of all tested force fields. Temperature and solvent models are reflected by color as shown in the legend. Quantum mechanical calculation results (black dots) are given for reference. Worst outliers are (molecules in A and force fields in B) are labeled.

We analyzed the obtained ensembles in two ways: by comparison of the conformational plots as φ, ψ, and ω torsion distributions in all the obtained frames (Figure 2 and Figure 3, and superimposed data in Supplementary Figure S1 for reference) and by the prediction of NOE values (Figure 4, Table 1, and Supplementary Tables S1–S7). The distribution of results agrees with the earlier observation [35] that second generation force fields, including GLYCAM, provided similar results in energy minimization of disaccharides, while older force fields yielded highly dispersed results. The remarkable exclusion had been MM3, which outperformed other old force fields, and was less sensitive to the medium permittivity. It should be noted that the above conclusion was made from energy calculation on a bulk set of disaccharide conformers, in vacuum, with various permittivity values and subsequent comparison of energies with crystal state DFT calculations.

In general-purpose force fields, as well as in Amber ff14SB, topology generation was a straightforward procedure that could easily be automated. In CHARMM and GLYCAM, manual manipulations with input files had to be applied for the generation of topologies of the unsupported residues (see Methods, Section 3.2). In case of CHARMM, we started from an existing topology of a residue structurally close to that under study. After that, we reassigned atom names, types and partial charges analogously to recognized residues, keeping the total charge of a fragment equal to the integer formal charge.

In case of GLYCAM, the approach above could be replaced by less labor intensive semi-automated procedure. Topology generation for GLYCAM was done by stock features of AmberTools [36]; however, calculation of the partial charges became a problem. In the original publication on GLYCAM-06 [6] atomic charges were ensemble averaged from restrained electrostatic potential (RESP) [37]/HF/6-31G* charges over 100–200 representative structures from a 50 to 100 ns long MD trajectory. This approach is anyway unacceptable for database filling due to its high resource cost, so we studied two simplified approaches: (1) single point RESP/6-31G* charges based on initial geometry followed by dynamics in Amber package; and (2) AM1-BCC charges [38] followed by dynamics in Gromacs package.

**Figure 5:**
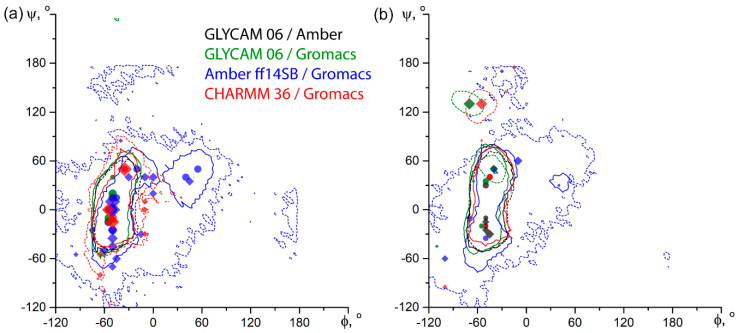
Comparison of energy plots for inter-glycosidic dihedrals of α-D-Man*p*-(1-3)-α-D-Man*p*-OMe **2** (**a**) and α-D-Rha*p*4N-(1-2)-α-D-Rha*p*4N-OMe **7** (**b**) obtained in dedicated carbohydrate force fields. Solid lines depict explicit TIP3P water simulations, dashed lines are for GB solvation model. Contours encircle an area within +2 kcal/mol from a global minimum. All minima are plotted by using symbols: circles for explicit TIP3P water simulations, and diamonds for GB solvation simulations. Circle area and diamond size correspond to 2 + log_10_ (minimum occupation related to total frame count, in percent). Colors reflect the force field and its software-dependent modification, as indicated in the legend.

On both α-D-Man*p*-(1-3)-α-D-Man*p*-OMe (**2**) and α-D-Rha*p*4N-(1-2)-α-D-Rha*p*4N-OMe (**7**), as representative examples of an uncharged and charged molecule, respectively, explicit solvent conformation maps obtained from the two schemes of charge assignment (Figure 5) were very close to each other. On the contrary, implicit solvent maps from Gromacs (i.e., in CHARMM, Amber, and GLYCAM force fields) showed auxiliary populated areas and were generally more diffuse than those from Amber (GLYCAM force field). Probably, this could be attributed to peculiarities of implementation of the implicit solvation in Gromacs, and Gromacs has stopped support of such solvent models since version 2019.1.

Table 1 shows root mean square deviation (RMSD) values for NOE comparison using different force fields on eleven models. For each force field, the best results from the two temperatures (300 and 1000 K) are given. For universal force fields, HCT solvation model was selected for presentation as it produced better results than Still. More details and calculation of RMSD are available in Supplementary Tables S1–S7; experimental conditions and other details on published NOEs are in Supplementary Table S8.

The top three methods revealed by ranking are shown in Table 2. According to quadratic ranking with absolute RMSD, MM3 with explicit water was the most accurate, DFT calculation occupied the second rank with virtually similar accuracy, and the third place was given to Glycam with explicit water. The results from ranking match those from a common sense, as each of the first two methods occupied the first rank twice, and the second rank once, while the sum of third ranks showed better accuracy of MM3. Usage of a linear scale left two best methods untouched, placing implicit solvent MM3 simulation to the third place. Usage of relative RMSD with linear scale rearranged top three methods, but the top-three list itself has not changed. Quadratic scale virtually equalized implicit solvent MM3 and MMFF at 1000 K as most accurate predictors outperforming MM3 with explicit solvent and DFT (fourth rank). Upon all ranking models, the gap between DFT and MM3 was not dramatic.

**Figure 6:**
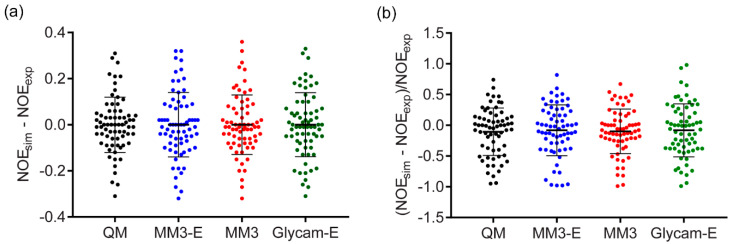
Distribution of individual NOE simulation errors from four methods providing generally best accuracy. “-E” stands for explicit solvent. Range bars show the RMSD value and the standard deviation. (**a**) Absolute individual NOE errors. (**b**) Relative individual NOE errors. Data from Supplementary Tables S1, S4-1, and S7.

There has been no clear answer published on whether the relative RMSD (Supplementary Table S9) was more adequate metrics than the absolute RMSD (Table 1) in NOE simulation accuracy tests. Pearson’s correlation coefficient between absolute and relative RMSDs is only 0.3. On the one hand, the normalized relative RMSD fits a common sense and was reported as a good estimator of NOE prediction power [46]. On the other hand, the normalized metrics tended to overestimate the value of errors, when they were low in absolute scale but experimental NOE was weak, and to ignore higher absolute error values when NOE itself was strong. For a deeper view, we compared distributions of individual NOE simulation errors (Figure 6) from four methods that showed generally better accuracy: DFT sampling, GLYCAM, and MM3 dynamics with explicit and implicit solvent models. As judged from these distributions, the choice of metrics virtually did not affect the quantitative estimation of a method accuracy. In a discussion below, we used absolute RMSD. The calculations and ranking based on relative RMSD is available in Supplementary Materials for reference.

With a few exceptions (CHARMM36, molecules **2**, **8**, **9**, **11**), ensembles obtained by implicit solvent molecular dynamics at 300K occurred inside the populated regions of 1000K distributions, as expected. For CHARMM36, this means that these molecules occupy a deep local minimum at 300K, however a global minimum is located elsewhere. Since only molecules **9** and **11** of the abovementioned ones required topology manipulation, this issue requires further attention. In most cases, ensembles with explicit solvent resembled those with implicit solvation at 300K, however exceptions exhibited a clear correlation with molecular peculiarities. **7**, **9**, and **11** contain residues, for which no standard parameters were present in the dedicated force fields. They displayed a visible φ-ψ distribution mismatch between explicit and implicit solvation models; for these molecules, a better performance of explicit solvent dynamics in NOE prediction was most remarkable (see Supplementary Tables S10 and S11). We suppose that explicit solvation compensated for the absence of torsion angle parameters in atypical residues.

Comparison of results from general purpose force fields (MM3, MMFF, and OPLS) obtained at high and low temperature showed that MM3 performed best of them with both implicit and explicit solvent models. For all molecules the distribution at 300 K occupied the area of greatest probability in a 1000 K distribution, except a single outlier (**9**, OPLS). Amber, CHARMM36, and GLYCAM population tended to display the same pattern but with some exceptions (Supplementary Table S11, upper block). In most samples, the predominant state of a molecule on a φ-ψ conformation map occupied minima different from conformations of the initial geometry (dots and vertical lines in Figure 2 and Figure 3), thus proving that results were not distorted by the initial geometry choice.

Explicit solvation drastically increases the number of atoms in simulation, thus affecting the computer resource cost. Taking into account that our intended further usage of a best method is very sensitive to computational performance due to a large number of objects to process, the choice of explicit solvation is reasonable only if it is proven to produce much more accurate results. In the case of MM3, the solvation model effect was contradictory: Explicit solvation improved the NOE prediction accuracy in four cases, deteriorated it in four other cases, and made no valuable change in three cases. The similar pattern was observed for Amber and CHARMM; introduction of explicit solvent improved the result only in two cases for CHARMM and five cases for Amber (Supplementary Table S11, lower block). GLYCAM 06 was designed especially for usage with explicit solvent model, and, expectedly, it showed better or at least not worse accuracy of results from explicit solvation. Another notable feature was demonstrated by explicit solvent simulations: In most cases, glycosidic dihedral distributions had a very similar visual appearance under different force fields; however, they yielded drastically different NOE accuracy (i.e., in **2**, **4**, **6**, **10**). This might result from differences in molecular motion, such as ring dynamics, which had no direct effect on φ-ψ Rhamachandran plots; however, it still needs to be addressed in further studies.

Although more convenient from the point of view of compatibility and potential automation, general-purpose force fields generally performed worse than the dedicated ones, except for MM3. The comparison of NOE simulation accuracy from dynamics in these force fields is available in Supplementary section “Comparison of General-Purpose Force Fields between Each Other” for reference. A special note should be made about MMFF, as it is most compatible with arbitrary structural features and, probably, most convenient in automated calculations due to wide support in programming libraries. In MMFF dynamics, the most populated conformations had *inter-*glycosidic torsions at 300 K close to that at 1000 K for a few molecules only. It can be concluded that a high temperature is critical for obtaining sufficient conformational space in MMFF force field, while MM3 and OPLS-aa achieve enough transitional freedom at 300 K. Extra molecular dynamics calculations at 400 and 500 K were carried out for one exemplary disaccharide to analyze the temperature effect on the conformational space (Supplementary Figure S2 and Supplementary Table S12). 400 K appeared to be the minimal temperature to achieve a representative ensemble in MMFF and OPLS-aa, while MM3 did not have this limitation. In all force fields tested, high temperature led to pyranose “de-chairing” and diffusion of population to non-predominant conformations of monosaccharides (Supplementary Figures S3 and S4). In implicit solvent simulations, this behavior could be avoided by applying restraints on *intra-*glycosidic torsions. In explicit solvent simulation, usage of 1000 K made no sense, as expected due to moving of water molecules away from a disaccharide and a loss of solvation effects. As it was mentioned earlier, this behavior could be avoided by the usage of constant volume simulations; however, we chose to stay within a physically correct isothermal-isobaric (NPT) ensemble.

NOEs predicted from DFT-derived minima was still the closest to the experimental values. However, the difference was not dramatic: RMSD averaged over all molecules was 0.10 from DFT minima, 0.11–0.20 from full conformational space of MD trajectories with implicit solvent, and 0.11–0.26 from those with explicit solvent. On the other hand, even a limited sampling from a conformational space (from four to twelve [φ,ψ]-minima after clustering, see Supplementary Figure S5) required much more computer resources than molecular dynamics simulation in any molecular mechanic force field. Taking into account the purpose of this study, we concluded that, for database filling, the better accuracy of quantum mechanical calculations was not worth the computational costs.

The simulated NOEs, as well as their RMSD (simulated vs. experimental), are given in Supplementary Tables S1–S7. Figure 4 summarizes matching between the conformational plots and predicted NOEs. Panel A shows that DFT sampling and MM3 gave most accurate predictions. GLYCAM and Amber with explicit solvent performed little worse, and CHARMM and MMFF were clear outsiders.

Most problematic molecules, especially in implicit solvent and MMFF simulations, were **2**, **7** and **8** (Figure 4B), with the two latter being composed from an atypical residue (α-D-Rha*p*4NH_2_) only. Introduction of explicit water molecules helped in both cases, especially for **7**, which has a 1-2-linkage keeping residues spatially closer to each other, like in branched structural fragments. NOEs in molecules with an exocyclic linkage (**3**, **4**, **5**) could be predicted generally better (or equally good, for **3**) than NOEs in molecules with two rotational degrees of freedom of a glycosidic bridge. As NOE characteristic time is longer than a trajectory length, additional degree of freedom could improve accuracy by easier access to a global energy minimum. For molecules **1**, **2**, **3**, **4**, **9**, **10**, and **11,** at least one empirical force field with explicit solvent model outperformed the DFT sampling. Explicit solvation provided considerably more accurate NOE simulation than the implicit solvation at 300 K for four of eleven molecules. In the other seven cases, explicit solvation was not worse than the implicit one; however, its advantage was not obvious.

The computational performance of all molecular mechanic force fields in Amber and Gromacs did not differ significantly; for disaccharide dynamics, it ranged from one to four hours per every 25 nanoseconds of a trajectory per graphic processor unit (Nvidia Tesla K20m or Nvidia 2080Ti class). Surprisingly, models with implicit and explicit solvent did not show even a two-fold difference in execution time. This can be attributed to the insufficient parallelization capabilities in implicit-solvent calculations. As a qualitative estimation, quantum-mechanical calculations showed ten- to thousand-fold performance decrease. At present, it rendered it inapplicable for the purpose of massive simulations.

## 3. Materials and Methods

### 3.1. Selection of Objects and Starting Geometries

Disaccharides and their modifications, for which the quantitative equilibrium NOEs were reported, were used for the MD simulation (see Supplementary Table S8). Of published data found by keywords on WebOfScience and PubMed, we picked molecules containing representative structural features from various classes of carbohydrates (widespread constituents, uronic acids, furanoses, bonds with two or three torsions, atypical residues, etc.). Another requirement was that the data collection was well documented and performed at a temperature near 300 K, as steady-state NOE measurement.

Starting geometries (Supplementary Materials, section “Initial Geometries”) were generated by MMFF94 relaxation of inter-residue bridges with unconstrained initial geometry of monomers, corresponding to their predominant conformations (as implemented in CSDB modeler, http://csdb.glycoscience.ru/csdb2atoms.html) [47]. Prior to any molecular dynamics simulation, starting geometry for a single disaccharide molecule was energy-minimized with a 0.01 kcal/mol/Å gradient cutoff in the same force field as used for MD tests. For the dedicated force fields, molecules were further equilibrated, heated during preprocessing, and saved in the second frame in each trajectory.

### 3.2. Force Fields

Molecular dynamics simulations in the universal force fields (MM3-2000, MMFF94, and OPLS-AA) were performed, using TINKER 8.6.1 software [48] (https://dasher.wustl.edu/tinker/). Parameters for the OPLS-AA force field were calculated with freely available LigParGen [49] OPLS/CM1A parameter generator (http://zarbi.chem.yale.edu/ligpargen/); three molecule optimization iterations were chosen.

MD simulations in CHARMM36 force field [19,20] were performed by using GROMACS 2018.8 software [26]. To generate topologies, “Glycan reader & modeler” module of CHARMM-GUI parameter generator [50] was used. In case of inability to generate parameters automatically (namely for α-D-Glc*p*-(1-2)-β-D-Fru*f* glycoside linkage, α-D-Rha*p*4N, α-D-Fuc*p*NAc4N, and α-KDO-OAllyl), topology for the closest structural analogue was generated and further manipulated manually.

MD simulations in Amber ff14SB [18] (as an all-purpose force field; all molecules) and GLYCAM-06 (molecules containing non-parameterized residues α-D-Rha*p*4N, α-D-Fuc*p*NAc4N, and α-KDO-OAllyl) force fields were performed, using GROMACS 2018.8 software. To generate topologies, *antechamber* [51] program from AmberTools 19 package [36] was used for AM1-BCC [38] charge calculation and atom type assignment, followed by *tleap* program from the same package to generate Amber topology with GLYCAM or ff14SB parameters. Amber topologies were converted to GROMACS format, using *Acpype* [52,53].

In case of GLYCAM-06 force field, [6] which can be considered as Amber ff14SB extension for carbohydrates, topologies were constructed via Carbohydrate Builder module at http://glycam.org for disaccharides with constituents having a GLYCAM three-letter code naming and explicitly parameterized within the force field. Calculations were carried out in Amber12 [24].

### 3.3. Solvation Models

In the universal chemical force fields, HCT [54] and Still [55] models were used for implicit solvation. Of them, HCT showed better results and was used further. For the MM3 force field, a molecular dynamics simulation was also carried out with explicit water solvation (20 × 20 × 20 Å box of ca. 240 water molecules).

Calculations in the dedicated biomolecular force fields (Amber ff14SB [18], Charmm 36 [19,20], and GLYCAM 06 [6]) in Amber 12.0 [24,56] were conducted, using Generalized Born solvent model [57] and, additionally, explicit water box. For Amber, 10 Å minimal distance between a solute molecule and a bound of a periodic box was used. In three trials starting from 6Å, this water box was found as the smallest one enough to avoid minimization errors persistent through multiple iterations. For CHARMM and GLYCAM, periodic box size provided a 15 Å minimal distance between a solute molecule and a box face, typically at least 30 × 30 × 30 Å.

### 3.4. Simulation Parameters

MD simulations were performed by using 2 fs integration time-steps. P-LINCS [58] was employed to constrain bond lengths. Non-bonded interactions were truncated at 12 Å, and Particle Mesh Ewald algorithm [59] was used for long-range electrostatics. Temperature was controlled by using Berendsen thermostat [60], and a Parrinello–Rahman barostat [61] was used for constant pressure.

Calculations with explicit solvent were performed by using TIP3P [62] water model at 300 K, but not at 1000 K, as the latter is well above water-boiling temperature and requires switching to an NVT ensemble. Calculations with implicit solvent were performed at 300 and 1000 K for all molecules under study, and additionally at 400 and 500 K for selected molecules (**1**, α-D-Glc*p*-(1-2)-β-D-Fru*f* and **9**, α-D-Gal*p*A-(1-3)-α-D-Fuc*p*2NAc4NH2-OMe, see Supplementary Table S12).

In unrestrained 1000 K simulations with implicit solvation, model ring conformation underwent continuous transitions as expected for this high temperature. To stabilize pyranose rings, restrains on C1-C2-C3-C4 were applied. Details on ring puckering in constrained and unconstrained high-temperature simulations were exemplified on sucrose (Supplementary Figure S6).

MD trajectories had a length of 50 or 100 ns (see details in Table 1) with snapshots written each 2 ps, thus producing 25,000 or 50,000 structures (frames) per simulation. To prove a writing step of 2 ps is enough, molecular dynamics with geometries recorded each 0.05 ps was also carried out for one of disaccharides (**4**, α-D-Man*p*-(1-6)-β-D-Man*p*-OMe). Calculated NOEs were virtually identical to those obtained from 2 ps frame rate (see RMSD values in Supplementary Table S13).

### 3.5. DFT Calculations

Quantum mechanical processing of every trajectory frame is too resource-greedy to be used in multiple MD simulations. In order to obtain a realistic representation of the modeled conformation ensembles with minimal DFT calculations, all frames obtained in the MM3 force field at 300 K for each molecule were aligned by all non-hydrogen atoms and clustered using k-means algorithm as implemented in *NbClust* [63] package (distance = “Euclidean”, index = “silhouette”). All cluster counts between 25 and 50 were taken into consideration, and the cluster assignment corresponding to the optimal index was used at the next stage. Optimal number of clusters was 29 for **1**, 27 for **6**, 26 for **4** and **11**, and 25 for all other molecules. Structures closest (by Euclidean distance) to the cluster centers were used as initial geometries in DFT calculations. DFT calculations were performed at PBE0-D3 theory level [64,65] in def2-TZVP basis set [66] with water solvation effects included, using the solvation model based on density (SMD) [67] and standard convergence criteria of 1.5 × 10^−5^ Harthree/Bohr in Gaussian09 D.01 [68] software. PBE0 functional is known to provide accurate results in carbohydrate modeling [9,69] and was recently shown to be well-grounded in theory [70,71]. After optimization convergence, harmonic frequencies were computed at the same level of theory to ensure that located structures correspond to the expected types of stationary points (minima) and were subsequently utilized to compute quasi-harmonic [72,73] free energies, using *GoodVibes* program [74]. Boltzmann analysis was done by using the resulting free energies to estimate which conformations contribute the most to the NOEs via weighted averaging. Relative quasi-harmonic free energies of all located minima, their ratios in solution at 300 K and their 3D structures are provided in Supplementary Materials, section “DFT-Derived Minima”.

### 3.6. NOE Simulation and Comparison

All obtained geometries were analyzed by using R scripting language [75] with *tidyverse* [76] package. For NOE calculation, values for each pair of protons were computed separately for each frame, using the following equation [77]: $NOE_{a}\left(b\right)=r_{ab}^{-6}/2\underset{i\neq a}{\sum }r_{ai}^{-6},$ where *r* is H–H distance, *a* is an observed proton, and *b* is a saturated proton. Exchangeable protons (-OH, -NH_2_, and -COOH) were not counted in this summation. Then NOEs were averaged from these values in all frames: $NOE=\;\sum^{\;}NOE_{a}\left(b\right)/n,$ where *n* is a number of frames (typically 25,000). Prior to comparison, both experimental (Supplementary Table S8) and simulated NOEs were normalized to the sum of all NOEs observed upon saturation of a certain proton. This approach was undertaken to improve reliability of comparison between calculated and experimental values in terms of RMSD. When the sum of NOEs was presented, the simulation values were also validated by summing the indicated proton intensities. The simulated NOEs were compared with the experimental ones using root-mean-square deviation (RMSD) and relative RMSD according to the following formula [46]: $RMSD_{rel}=\sqrt{\sum^{\;}{\left((NOE_{i}-NOE_{iExp})/NOE_{iExp}\right)}^{2}/N,}$ where *N* is a number of NOEs observed in a molecule.

### 3.7. Ranking of NOE Simulation Results

The method score was derived from individual ranks of RMSD between calculated and experimental NOEs of individual molecules: 64 points for 1st rank, 32 points for 2nd rank, etc., and 1 point for 7th rank; 8th and lower ranks produce a zero addend; the sum of points produces the overall score. More details are available in Supplementary Table S10. This ranking scheme originates from a multi-agent knapsack problem [78] with eleven *voting molecules* and arbitrary *budget size*. If each molecule voted for a single best method only, the winner could not be revealed. To simplify the problem, we assigned points to the seven top methods, using quadratic and linear models. In a quadratic model, top rank gives more points than all other ranks together. In a linear model we used progression value 9, as it gave the highest rank (63) closest to that of quadratic model (64) (see Supplementary Table S10, right side).

### 3.8. Presentation

All plots were obtained with plotnine [79] and OriginPro (OriginLab Corp, Northampton, MA, USA) [80].

A Microsoft Excel 2010 template (Supplementary Figure S7, and section “Trajectory Visualization” in Supplementary Materials) was designed for quick visualization of abundance and energy maps; their projections on ψ, φ, and ω axes and on φψ, φω, and ψω planes; dihedral scatter data; and transition rate. The input data to be pasted into the template are dyads or triples of torsions, one per trajectory frame. Excel 3D Scatter Plot 2.1 (https://www.doka.ch/Excel3Dscatterplot.htm) scripts were used for [ψ,φ,ω]-scatter.

## 4. Conclusions

The ideal saccharide conformation prediction method for massive and unmanned database filling should fit the following criteria: simulation accuracy, computational performance, and easiness of automation. We probed the simulation accuracy of methods by comparison of predicted and experimental NOEs of model disaccharides, and selected four candidates with maximal accuracy: quantum-mechanic sampling, MM3 molecular dynamics with explicit and implicit solvent, and GLYCAM with explicit solvent. Although accuracy of MM3 dynamics with explicit and implicit solvent models did not differ dramatically, we gave preference to explicit water simulations, as they were more chemically correct.

In comparison to DFT sampling, which required much more computational resources, execution-time difference between individual MD methods could be neglected. Moreover, DFT required initial geometries generated by MD anyway.

From the point of view of automation, CHARMM was the least convenient, as it required manual adjustment of parameter files for atypical residues. Meanwhile, almost half of the residue types in bacterial saccharides are atypical in this context [81]. Amber and GLYCAM allowed easier implementation of the calculation pipeline; however, it was impossible to avoid manual operations fully, especially for charged molecules. Older general-purpose force fields outperformed the dedicated ones in this viewpoint, as they allowed fully automatic parametrization.

Due to a combination of the above criteria, we have chosen MM3 molecular dynamics with explicit water at 300 K as most favorable method for bulk conformational computations of disaccharides for the automated database filling.

## Figures and Tables

**Table 1:** Deviations between experimental NOEs and NOEs calculated from MD trajectories in different force fields. Absolute RMSD is provided for all observed proton-proton correlations. Average values are arithmetic means from eleven molecules, calculated separately on a sampling of all observed NOEs, and transglycosidic NOEs only.

#	Force Field	MM3 2000		MMFF94	OPLS aa	GLYCAM 06		CHARMM 36		Amber ff14SB		DFT ^a^	NOE Reference
	solvation model ^b^	HCT	TIP3P ^c^	HCT	HCT	GB	TIP3P ^d^	GB	TIP3P ^d^	GB	TIP3P ^d^	SMD	
	Temperature, K ^e^	300	300	1000	300	300	300	300	300	300	300	300	
**1**	α-D-Glc*p*-(1-2)-β-D-Fru*f*	0.17	0.22	0.23	0.23	0.18	0.20	0.15	0.28	0.17	0.15	0.18	[39]
**2**	α-D-Man*p*-(1-3)-α-D-Man*p*-OMe	0.09	0.13	0.30	0.30	0.05	0.03	0.24	0.37	0.11	0.18	0.05	[40]
**3**	α-D-Man*p*-(1-6)-α-D-Man*p*-OMe	0.14	0.14	0.22	0.22	0.17	0.18	0.15	0.03	0.15	0.15	0.17	[40]
**4**	α-D-Man*p*-(1-6)-β-D-Man*p*-OMe	0.03	0.02	0.10	0.10	0.06	0.07	0.04	0.51	0.04	0.06	0.07	[41]
**5**	β-D-Glc*p*NAc-(1-6)-α-D-Man*p*-OMe	0.04	0.05	0.05	0.05	0.05	0.05	0.12	0.23	0.05	0.04	0.03	[41]
**6**	β-D-Glc*p*NAc-(1-2)-α-D-Man*p*-OMe	0.09	0.08	0.07	0.07	0.13	0.13	0.08	0.20	0.13	0.15	0.08	[41]
**7**	α-D-Rha*p*4N-(1-2)-α-D-Rha*p*4N-OMe	0.10	0.07	0.29	0.29	0.28 ^f^	0.15	0.41	0.13	0.09	0.15	0.04	[42]
**8**	α-D-Rha*p*4N-(1-3)-α-D-Rha*p*4N-OMe	0.16	0.10	0.32	0.32	0.16 ^f^	0.13	0.17	0.26	0.18	0.18	0.07	[42]
**9**	α-D-Gal*p*A-(1-3)-α-D-Fuc*p*NAc4N-OMe	0.16	0.16	0.18	0.18	0.29 ^f^	0.20	0.31	0.31	0.32	0.17	0.18	[43]
**10**	α-L-Rha*p*-(1-2)-α-L-Rha*p*-OMe	0.10	0.10	0.16	0.16	0.14	0.14	0.21	0.38	0.16	0.14	0.13	[44]
**11**	α-KDO-(2-4)-α-KDO-OAllyl	0.15	0.16	0.25	0.25	0.20 ^f^	0.08	0.25	0.18	0.20	0.12	0.10	[45]
	average (all NOEs) ^g^	0.11 (0.33)	0.11 (0.33)	0.20 (0.60)	0.13 (0.41)	0.15 (0.47)	0.12 (0.40)	0.19 (0.64)	0.26 (0.92)	0.15 (0.53)	0.13 (0.42)	0.10 (0.36)	
	average (transglycosidic NOEs)	0.10	0.10	0.22	0.10	0.14	0.11	0.18	0.21	0.14	0.13	0.10	
	overall rank ^h^	4	1	5	11	8	3	10	7	12	6	2	
	software ^i^	Tinker		Tinker	Tinker	Amber, Gromacs		Gromacs		Gromacs		Gaussian	
	supplementary table	S1	S1	S2	S3	S4-1	S4-1	S5	S5	S6	S6		
	trajectory length, ns	50	50	50	50	100	100	50	50	50	50		

^a^ Theory level and basis set: PBE0-D3/def2-TZVP (Triple-Zeta Valence Polarized). ^b^ For universal force fields, HCT solvation model was selected for presentation as it produced better results than Still. ^c^ 20 × 20 × 20 Å water box. ^d^ 30 × 30 × 30 Å water box. ^e^ In implicit solvent calculations, 300 and 1000 K were tested; better results (regarding absolute and relative RMSD) were presented. ^f^ Gromacs was used for charged molecules instead of default Amber, as Gromacs topologies are more convenient for manipulation. ^g^ Absolute RMSD (regular font). Average values for relative RMSD are given in italics for reference. Full data on relative RMSD are in Supplementary Table S9. ^h^ Absolute RMSD, quadratic ranking (see text). ^i^ Software versions: Tinker 8.6.1, Amber 12.0, Gromacs 2018.8, Gaussian 09. DFT = Density Functional Theory; HCT = Hawkins, Cramer and Truhlar; TIP3P = Transferable Intermolecular Potential 3 Point; GB = General Born; SMD = Solvation Model based on Density.

**Table 2:** Top three methods in different ranking schemes.

Ranking Scheme	1st Rank	2nd Rank	3rd Rank
Absolute RMSD, quadratic scale	MM3, explicit	DFT	Glycam, explicit
Absolute RMSD, linear scale	MM3, explicit	DFT	MM3, implicit
Relative RMSD, quadratic scale	MM3, implicit	DFT	MM3, explicit
Relative RMSD, linear scale	MM3, implicit	MM3, explicit	DFT

## Supplementary Materials

Supplementary materials can be found at https://www.mdpi.com/1422-0067/21/20/7626/s1.

## Abbreviations

CSDB	Carbohydrate Structure Database
DFT	density functional theory
GB	general born
HCT	Hawkins, Cramer and Truhlar
MD	molecular dynamics
NMR	nuclear magnetic resonance
NOE	nuclear Overhauser effect
NPT	isothermal-isobaric (substance (N), pressure, and temperature are conserved)
QM	quantum mechanics
PBE	Perdew, Burke, and Ernzerhof
RESP	restrained electrostatic potential
RMSD	root mean square deviation
SMD	*solvation model* based on density
TIP3P	transferable intermolecular potential 3 point
TZVP	triple-zeta valence polarized
