# Supplementary material for: Comparison of Methods for Bulk Automated Simulation of Glycosidic Bond Conformations
Source: Int J Mol Sci. 2020 Oct 15;21(20):7626. doi: 10.3390/ijms21207626 (PMC7589101; doi:10.3390/ijms21207626)
Supplement: Supplementary file 1 [file ijms-21-07626-s001.zip › Supplementary_Materials.doc]

**Supplementary Materials**

**Comparison of methods for bulk automated simulation of glycosidic bond conformations**

V.S. Stroylov^1,2^, M.V. Panova^1^, Ph. V. Toukach^1,2,*^

*^1^ N.D. Zelinsky Institute of Organic Chemistry, Russian Academy of Sciences, 119991 Russia, Moscow, Leninsky pr. 47
^2^ National Research University Higher School of Economics (HSE), Chemical Faculty, 20 Myasnitskaya Street, 101000, Moscow, Russian Federation*

^*^ To whom correspondence should be addressed: [netbox@toukach.ru](mailto:netbox@toukach.ru)

# Contents

Calculated NOEs (references to Tables S1-S7) *	2

Experimental NOEs, minor tests, ranking (references to Table S8, and Tables S10-S13) *	2

Deviations between experimental NOEs and NOEs calculated from MD trajectories (Table S9)	2

Combined conformational plots (Fig. S1)	4

Initial geometries (references to MOL files)	6

Comparison of general-purpose force fields between each other	7

Temperature effect on conformational space (Fig. S2)	7

Full trajectories (reference to archive)	7

Conformations of six-membered carbohydrate rings (Fig. S3, Fig. S4)	8

DFT-derived minima (Fig. S5)	10

Conformation of pyranose rings (Fig. S6)	11

Trajectory visualizer (Fig. S7, reference to Microsoft Excel macros)	12

References	13

* For tables S1-S8 and S10-S13, please use a supplementary Mirosoft Excel file to access the data.

Calculated NOEs

*File: Supplementary_tables_S1-S8_and_S10-S13.xlsx*

NOEs calculated from trajectories in various force fields at 300K and 1000K, with implicit and explicit solvation models. Absolute and relative RMSD (for each molecule, and averaged), is calculated in spreadsheets separately for all protons that have NOE and for *inter-*glycosidic protons only.

**Table S1** (tab MM3): MM3 2000

**Table S2** (tab MMFF): MMFF94

**Table S3** (tab OPLS): OPLS-AA

**Table S4-1** (tab Glycam): GLYCAM06

**Table S4-2** (tab Glycam CHRG): GLYCAM06 of charged molecules, alternative parametrization

**Table S5** (tab Charmm): CHARMM with C36

**Table S6** (tab Amber): AmberFF

**Table S6-1** (tab Ambers HC): Ambers as implemented in HyperChem 8 (found erroneous; not used)

**Table S7** (tab QM): DFT calculation (PBE0-D3/def2-TZVP, SMD)

# Experimental NOEs, minor tests, ranking

*File: Supplementary_tables_S1-S8_and_S10-S13.xlsx*

**Table S8** (tab Experimental NOE): observed and relative NOE data and experimental conditions from literature

**Table S10** (tab Method ranking): Ranking of methods regarding NOE simulation accuracy

**Table S11** (tab Implicit vs. explicit): Is population density behavior expected?

**Table S12** (tab mol01 temperature): NOEs of mol01 and mol09 at different temperatures in MM3 MD

**Table S13** (tab mol04 printstep): NOEs of mol04 at different printstep in MM3 MD

**Table S9.** Deviations between experimental NOEs and NOEs calculated from MD trajectories in different force fields. **Relative** RMSD is provided for all observed proton-proton correlations. Average values are arithmetic means from eleven molecules, calculated separately on a sampling of all observed NOEs, and transglycosidic NOEs only.

| # | force field | MM3 2000 | | MMFF94 | OPLS aa | GLYCAM 06 | | CHARMM 36 | | Amber ff14SB | | DFT ^a^ | NOE ref. |
| --- | --- | --- | --- | --- | --- | --- | --- | --- | --- | --- | --- | --- | --- |
|  | solvation model ^b^ | HCT | TIP3P ^c^ | HCT | HCT | GB | TIP3P ^d^ | GB | TIP3P ^d^ | GB | TIP3P ^d^ | SMD |  |
|  | temperature ^e^ | 300 | 300 | 1000 | 300 | 300 | 300 | 300 | 300 | 300 | 300 | 300 |  |
| 1 | α-D-Glc*p*-(1-2)-β-D-Fru*f* | 0.45 | 0.54 | 0.70 | 0.79 | 0.60 | 0.66 | 0.60 | 0.66 | 0.66 | 0.49 | 0.46 | *[du Penhoat et al., 1991]* |
| 2 | α-D-Man*p*-(1-3)-α-D-Man*p*-OMe | 0.26 | 0.25 | 0.54 | 0.16 | 0.21 | 0.18 | 0.73 | 0.73 | 0.79 | 0.37 | 0.34 | *[Brisson and Carver, 1983]* |
| 3 | α-D-Man*p*-(1-6)-α-D-Man*p*-OMe | 0.43 | 0.41 | 0.46 | 0.48 | 0.50 | 0.52 | 0.43 | 0.12 | 0.46 | 0.45 | 0.51 | *[Brisson and Carver, 1983]* |
| 4 | α-D-Man*p*-(1-6)-β-D-Man*p*-OMe | 0.26 | 0.18 | 1.55 | 0.35 | 0.33 | 0.42 | 0.30 | 2.56 | 0.30 | 0.41 | 0.48 | *[Cumming and Carver, 1987]* |
| 5 | β-D-Glc*p*NAc-(1-6)-α-D-Man*p*-OMe | 0.19 | 0.31 | 0.50 | 0.46 | 0.34 | 0.25 | 0.35 | 0.69 | 0.42 | 0.30 | 0.32 | *[Cumming and Carver, 1987]* |
| 6 | β-D-Glc*p*NAc-(1-2)-α-D-Man*p*-OMe | 0.36 | 0.38 | 0.31 | 0.37 | 0.47 | 0.47 | 0.94 | 0.69 | 0.44 | 0.51 | 0.30 | *[Cumming and Carver, 1987]* |
| 7 | α-D-Rha*p*4N-(1-2)-α-D-Rha*p*4N-OMe | 0.21 | 0.20 | 0.22 | 0.29 | 0.46 ^f^ | 0.38 | 0.72 | 0.36 | 0.15 | 0.37 | 0.13 | *[Peters et al., 1990]* |
| 8 | α-D-Rha*p*4N-(1-3)-α-D-Rha*p*4N-OMe | 0.46 | 0.42 | 0.59 | 0.52 | 0.52 ^f^ | 0.53 | 0.65 | 0.60 | 0.71 | 0.55 | 0.33 | *[Peters et al., 1990]* |
| 9 | α-D-Gal*p*A-(1-3)-α-D-Fuc*p*NAc4N-OMe | 0.48 | 0.47 | 0.54 | 0.55 | 0.66 ^f^ | 0.55 | 0.70 | 0.69 | 0.70 | 0.49 | 0.50 | *[Bock et al., 1990]* |
| 10 | α-L-Rha*p*-(1-2)-α-L-Rha*p*-OMe | 0.33 | 0.30 | 0.45 | 0.38 | 0.38 | 0.38 | 0.53 | 0.71 | 0.45 | 0.34 | 0.35 | *[Mamyan et al., 1988]* |
| 11 | α-KDO-(2-4)-α-KDO-OAllyl | 0.53 | 0.68 | 0.80 | 0.52 | 0.65 ^f^ | 0.45 | 1.68 | 1.61 | 0.73 | 0.62 | 0.51 | *[Haselhorst et al., 1999]* |
|  | average (all observed NOEs) | **0.36** | **0.37** | **0.61** | **0.44** | **0.47** | **0.43** | **0.69** | **0.86** | **0.53** | **0.44** | **0.38** |  |
|  | software ^g^ | Tinker | | Tinker | Tinker | Amber, Gromacs | | Gromacs | | Gromacs | | Gaussian |  |
|  | supplementary table | S1 | S1 | S2 | S3 | S4-1 | S4-1 | S5 | S5 | S6 | S6 |  |  |
|  | trajectory length, ns | 50 | 50 | 50 | 50 | 100 | 100 | 50 | 50 | 50 | 50 |  |  |

a Theory level and basis set: PBE0-D3 / def2-TZVP.

b For universal force fields, HCT solvation model was selected for presentation as it produced better results than Still.

c 20x20x20 Å water box.

d 30x30x30 Å water box.

e Upon 300K and 1000K tested, better results were presented.

f Gromacs was used for charged molecules instead of default Amber, as Gromacs topologies are more convenient for manipulation.

g Software versions: Tinker 8.6.1, Amber 12.0, Gromacs 2018.8, Gaussian 09.

# Combined conformational plots

**Fig. S 1.** Superimposed glycosidic bond conformation plots:

Blue = 300K, implicit water

Red = 1000K, implicit water

Cyan = 300K, explicit water

Columns are eleven molecules under study; rows are force fields as indicated on the right. Dihedrals are defined as φ = H1–C1–O–Cx (C1–C2–O–C4' for α-Kdo-(2-4)-α-Kdo-OAllyl) and ψ = C1–O–Cx–Hx (C1–O–C2'–C1' for α-D-Glc*p*-(1-2)-β-D-Fru*f*), ω = Cx–Cx–C(x+1)‑H(*pro-S*).

Blue (300K) and red (1000K) dots (φ, ψ) and lines (ω) stand for starting geometries after minimization. The density plots were obtained from full MD trajectories. Contour levels denote equal difference in density of sampling.

RMSD between experimental and calculated NOE averaged on all interacting protons is provided in each plot:

Upper left corner (black) = 300K, implicit water

Lower left corner (green) = 300K, explicit water

*(figure is on the next page)*

# Initial geom**e**tries

*File: initial_geometries.zip*

Initial geometries used for molecular dynamics calculations obtained by MMFF94 relaxation of inter-residue bridges with unconstrained initial geometry of monomers, corresponding to their predominant conformations (as implemented in Carbohydrate Structure Database modeler, <http://csdb.glycoscience.ru/csdb2atoms.html>).

**mol1.mol**: α-D-Glc*p*-(1-2)-β-D-Fru*f*

**mol2.mol:** α-D-Man*p*-(1-3)-α-D-Man*p*-OMe

**mol3.mol**: α-D-Man*p*-(1-6)-α-D-Man*p*-OMe

**mol4.mol**: α-D-Man*p*-(1-6)-β-D-Man*p*-OMe

**mol5.mol**: β-D-Glc*p*NAc-(1-6)-α-D-Man*p*-OMe

**mol6.mol**: β-D-Glc*p*NAc-(1-2)-α-D-Man*p*-OMe

**mol7.mol**: α-D-Rha*p*4NH_2_-(1-2)-α-D-Rha*p*4NH_2_-OMe

**mol8.mol**: α-D-Rha*p*4NH_2_-(1-3)-α-D-Rha*p*4NH_2_-OMe

**mol9.mol**: α-D-Gal*p*A-(1-3)-α-D-Fuc*p*2NAc4NH_2_-OMe

**mol10.mol**: α-L-Rha*p*-(1-2)-α-L-Rha*p*-OMe

**mol11.mol**: α-KDO-(2-4)-α-KDO-Oallyl

**smiles.csv:** SMILES strings and CSDB Linear strings for molecules 01-11

# Comparison of general-purpose force fields between each other

MM3 performed best in NOE prediction for seven of eleven molecules. MMFF was the worst one in nine of eleven cases, and for five molecules (**1**, **2**, **7**, **9**, **10**) this behavior coincides with a poor ensemble. However, MMFF was the best for the other two molecules (**5**, **6**). OPLS-aa was the best force field for four molecules. It performed better than MM3 for three molecules (**2**, **8**, **11**), which was likely because MM3 recounted the existence of these molecules in a higher-energy region (MM3 equalizes all the regions in conformational plots more than OPLS-aa). It is noteworthy that for molecule **7** (α-D-Rha*p*4NH_2_-(1-2)-α-D-Rha*p*4NH_2_-OMe) MM3 and OPLS-aa were very close in NOE prediction, however, they produced different conformation maps: OPLS-aa ensembles occupied two regions approximately uniformly, while MM3 gave preference to one of them. However, RMSD derived upon sampling of all NOEs was different from RMSD from transglycosidic NOEs only, in the case of OPLS-aa but not MM3.

# Temperature effect on conformational space

**Fig. S 2.** Comparison of α-D-Gal*p*A(1-3)-α-D-Fuc*p*NAc4N(1-OMe (**9**) conformation space width at different MD temperatures. Dihedrals are defined as H1–C1–O–Cx (φ) and C1–O–Cx–Hx (ψ). Levels are linearly distributed between zero and 0.0001 density from abundance distribution over 25000 frames of MM3 (HCT) MD simulation.

# Conformations of six-membered carbohydrate rings

**Fig. S 3.** Conformations of six-membered carbohydrate rings (IUPAC definition according to Whitfield *[Bérces et al., 2001]*). Rings of donor residues are depicted (ones on the left in CSDB Linear notation).

**Fig. S 4.** Conformations of six-membered carbohydrate rings (IUPAC definition according to Whitfield *[Bérces et al., 2001]*). Rings of acceptor residues are depicted (ones on the right in CSDB Linear notation).

# DFT-derived minima

*File: DFT_optimized.xyz*

Relative quasi-harmonic free energies of all located minima, their ratios in water solution at 300K, and corresponding 3D structures (atom coordinates). Minima locations and energies are summarized in Fig. S 5.

**Fig. S 5.** [φ,ψ]-minima obtained by clustering and DFT calculation. The outermost density plot contours from each force field are given as dashed lines for reference; the contour color code is in the legend.

# Conformation of pyranose rings

At 1000K with implicit solvation model, stabilization of a pyranose ring in 4C1 conformation required restraints on two dihedrals, namely C1-C2-C3-C4 and С4-С5-О5-С1. Restraint on C1-C2-C3-C4 only did not fully stabilize a ring (Fig. S 6 B). Adding explicit water in 1000K simulations led to strong puckering even when high force constants were applied.

-100

-80

-60

-40

-20

0

20

40

60

80

100

-100

-80

-60

-40

-20

0

20

40

60

80

100

С1-C2-C3-C4

1

2

3

4

5 ns

С1-O5-C5-C4

1

2

3

4

5 ns

A.

С1-C2-C3-C4

1

2

3

4

5 ns

С1-O5-C5-C4

1

2

3

4

5 ns

B.

-62

-60

-58

-56

-54

-52

-50

-48

-46

-80

-60

-40

-20

0

20

40

60

80

**Fig. S 6.** Unrestrained (A) and C1-C2-C3-C4- restrained (B) pyranose ring puckering during first 5ns of MD simulation of sucrose (mol 01) in GLYCAM with GB solvation model at 1000K. Red lines show average values in one or two predominant states.

# Full trajectories

Raw molecular dynamics trajectories (4 Gb archive) are available at Zonedo:

*DOI: 10.5281/zenodo.4050512*

*URL: https://zenodo.org/record/4050512*

*Title: Comparison of methods for bulk automated simulation of glycosidic bond conformations - MD trajectory data*

File and folder names within the archive are self-explanatory, and encode a force field, molecule, trajectory length, temperature, and solvent model.

# Trajectory visualizer

*File: trajectory_visualization.zip (XLSM file in a zip archive)*

A Microsoft Excel 2010 template (Fig. S2) for quick visualization of abundance and energy maps, their projections on ψ, φ, and ω axes and on φψ, φω, and ψω planes, dihedral scatter data, and transition rate. The input data to be pasted into the template are duples or triples of torsions, one per trajectory frame. The template can load for long, please keep patience.

**Fig. S 7.** Microsoft Excel template for trajectory visualization.

# References

1.	du Penhoat, C.H., Imberty, A., Roques, N., Michon, V., Mentech, J., Descotes, G., Perez, S. Conformational behavior of sucrose and its deoxy analog in water as determined by NMR and molecular modeling*.* *Journal of the American Chemical Society*, 1991. **113**(10): 3720-3727. DOI: 10.1021/ja00010a014.

2.	Brisson, J.R., Carver, J.P. Solution conformation of alpha D(1-3)- and alpha D(1-6)-linked oligomannosides using proton nuclear magnetic resonance*.* *Biochemistry*, 1983. **22**(6): 1362-1368. DOI: 10.1021/bi00275a007.

3.	Cumming, D.A., Carver, J.P. Virtual and solution conformations of oligosaccharides*.* *Biochemistry*, 1987. **26**(21): 6664-6676. DOI: 10.1021/bi00395a016.

4.	Peters, T., Brisson, J.-R., Bundle, D.R. Conformational analysis of key disaccharide components of Brucella A and M antigens*.* *Canadian Journal of Chemistry*, 1990. **68**(7): 979-988. DOI: 10.1139/v90-154.

5.	Bock, K., Lönn, H., Peters, T. Conformational analysis of a disaccharide fragment of the polysaccharide antigen of Streptococcus pneumoniae type 1 using n.m.r. spectroscopy and HSEA calculations*.* *Carbohydrate Research*, 1990. **198**(2): 375-380. DOI: 10.1016/0008-6215(90)84309-i.

6.	Mamyan, S.S., Lipkind, G.M., Shashkov, A.S., Bairamova, N.E., Nikolaev, A.V., Kochetkov, N.K. Nuclear Overhauser Effect and Conformational States of Glycosyl-(1→2)- and -(1→3)-Rhamnosides in Aqueous Solution*.* *Russian Journal of Bioorganic Chemistry*, 1988. **14**(2): 205-215.

7.	Haselhorst, T., Espinosa, J.F., Jimenez-Barbero, J., Sokolowski, T., Kosma, P., Brade, H., Brade, L., Peters, T. NMR experiments reveal distinct antibody-bound conformations of a synthetic disaccharide representing a general structural element of bacterial lipopolysaccharide epitopes*.* *Biochemistry*, 1999. **38**(20): 6449-6459. DOI: 10.1021/bi982984z.

8.	Bérces, A., Whitfield, D.M., Nukada, T. Quantitative description of six-membered ring conformations following the IUPAC conformational nomenclature*.* *Tetrahedron*, 2001. **57**(3): 477-491. DOI: 10.1016/s0040-4020(00)01019-x.
